# Passive Immunotherapy of Cynomolgus Monkeys with Anti-Rotavirus IgY

**DOI:** 10.3390/pharmaceutics16091149

**Published:** 2024-08-30

**Authors:** Gentil Arthur Bentes, Juliana Rodrigues Guimarães, Eduardo de Mello Volotão, Natália Maria Lanzarini, Alexandre dos Santos da Silva, Noemi Rovaris Gardinali, Renato Sergio Marchevsky, José Paulo Gagliardi Leite, Jaqueline Mendes de Oliveira, Marcelo Alves Pinto

**Affiliations:** 1 Laboratório de Desenvolvimento Tecnológico em Virologia, Instituto Oswaldo Cruz, Fiocruz, Rio de Janeiro 21040-360, RJ, Brazil; 2 Laboratório de Virologia Comparada e Ambiental, Instituto Oswaldo Cruz, Fiocruz, Rio de Janeiro 21040-360, RJ, Brazil; 3 Laboratório de Ensaios Pré-Clínicos, Instituto de Tecnologia em Imunobiológicos, Fiocruz, Rio de Janeiro 21040-360, RJ, Brazil

**Keywords:** immunoglobulin Y, human rotavirus Group A, passive immunotherapy, cynomolgus monkeys

## Abstract

Immunoglobulins Y (IgY) purified from egg yolks of hens represents an attractive, cost-effective alternative for the development of new diagnostic and therapeutic platforms. In this study, we evaluated the therapeutic efficacy of rotavirus-specific IgY in a cynomolgus monkey (*Macaca fascicularis*) model. Animals were experimentally infected with human rotavirus Group A (RVA), the most common cause of severe acute diarrhoea among young children worldwide. Animals were administered human RVA (3.1 × 10^7^ FFU/mL) by oral gavage, challenged with 2.5 mg of anti-RVA IgY orally, and monitored for five days according to clinical, haematological and biochemical parameters; serum electrolyte levels; viral shedding; and histopathological changes. Immunotherapy with anti-RVA IgY had a protective effect against severe rotavirus-induced enteritis in four of the ten treated monkeys, as evidenced by histopathological findings. Although only one animal had diarrhoea, all but one exhibited virus shedding regardless of the treatment.

## 1. Introduction

Rotavirus acute gastroenteritis is a leading cause of diarrhoea among children, mainly in developing countries, and accounts for 80% of rotavirus-related deaths worldwide [1,2]. In Brazil, rotavirus morbidity and mortality in children under five years have substantially decreased since monovalent (Rotarix^®^, RV1, GlaxoSmithKline, Wavre, Belgium) and pentavalent (RotaTeq^®^, RV5, Merck & Co., North Wales, PA, USA) vaccines were included in the national childhood immunisation program [3]. However, heterotypic strains can be selected because the virus escapes from the host immune system in vaccinated individuals against the main circulating genotypes [4,5].

The management of rotavirus acute gastroenteritis relies on oral and/or intravenous rehydration to relieve symptoms and signs and restore physiological functions [6]. In general, either oral or intravenous rehydration therapy is the primary treatment. The oral route is preferred when mild to moderate dehydration occurs in children with acute diarrhoea [7,8,9]. However, for every twenty-five children orally treated, one will fail and require intravenous rehydration [7].

Local passive immunity is considered the most efficient strategy to protect the intestinal mucosa following rotavirus infection, and neutralising monoclonal antibodies have been used for this purpose [10]. However, chicken egg yolk rotavirus-specific IgY can be a cost-effective alternative to prevent and control this disease [11,12,13]. Some IgY studies against human and animal viruses have already been published [14,15]. A strong, active immune response against bovine rotavirus (BRV) and human rotavirus (HRV), purified from chicken egg yolk, was demonstrated in calves orally treated with BRV-specific immunoglobulin Y (IgY) and in neonatal gnotobiotic piglets treated with HRV-specific IgY, respectively [16,17]. Indeed, IgY technology has been applied as a passive immunotherapy or prophylaxis against several pathogens [18,19,20,21,22,23,24,25]. However, there is no published literature describing the use of IgY in nonhuman primates to treat rotavirus. The single article on immunotherapy with IgY in monkeys focused on staphylococcal enterotoxin B (SEB). All the rhesus monkeys that were treated survived lethal SEB aerosol exposure [26].

The stability of IgY in the gastrointestinal tract and its safety for treating gastrointestinal diseases are well documented [24,27]. Inhibition of rotavirus adhesion to enterocytes has been demonstrated in vivo [28]. In a previous study by our group, serial dilutions of a chicken anti-RVA IgY antibody were incubated with 3.1 × 10^7^ FFU/mL (fluorescent focus units/mL) RVA to confirm its ability to block virus adhesion to MA-104 rhesus monkey foetal kidney cells in vitro [29]. In the present study, we evaluated the therapeutic efficacy of the purified anti-RVA-IgY agent, which was orally and intravenously administered to cynomolgus monkeys experimentally infected with human RVA.

## 2. Materials and Methods

### 2.1. Monkeys

Fourteen clinically healthy cynomolgus monkeys (*Macaca fascicularis*), ranging in age from 10 months to 27 years and weighing from 1.20 to 6.65 kg (Table 1), were pre-screened and tested negative for RVA via serological and molecular assays during the quarantine period. All animals were obtained from the Primatology Sector, Institute of Science and Technology of Biomodels (ICTB) of the Oswaldo Cruz Foundation (Fiocruz), Rio de Janeiro, RJ, Brazil. The animals were housed at an animal biosafety level 2 facility during quarantine and throughout the experiment. The animals were individually housed in stainless steel squeeze back cages (25.098″ width × 31.903″ height × 30.658″ depth) in climate-controlled rooms (temperature of 23 ± 1 °C and humidity of 55 ± 5%) with 12 h light/12 h dark cycles and were fed daily with commercial primate diet supplements, fresh fruits, and vegetables. Water was provided ad libitum. All the animals had health certificates, which guaranteed the absence of infectious diseases. A serological survey confirmed that they were free of simian immunodeficiency virus (SIV) and simian type D retrovirus (SRV/D) [30]. The susceptibility of this nonhuman primate (NHP) model to rotavirus infection has been confirmed previously by our research group [31]. Environmental enrichment programs were offered throughout the study in the form of audio-visual (audios with forest themes) and tactile enrichment (toys such as hanging balls).

The Ethics Commission on Animal Use (CEUA) approved the experimental protocol (CEUA-Fiocruz LW-35/11). The monkeys were examined twice daily by veterinarians and technicians for clinical signs of anorexia, dehydration, emesis, and diarrhoea, which are typical signs associated with RVA infection. Haematological analysis, weight loss, and fever (≥38.5 °C) were always performed once a day during the early morning. The primary endpoints of severe gastroenteritis include debilitating diarrhoea, rapid weight loss (20% reduction in individual body weight before infection) and lethargy [32]. All clinical procedures were performed under anaesthesia, and all efforts were made to minimise painful procedures. The study protocol was conducted in strict accordance with the recommendations of the Guide for Care and Use of Laboratory Animals of the Brazilian Society of Science in Laboratory Animals (SBCAL) and the National Council for the Control of Animal Experimentation [33,34]. The staff has recognised experience in NHP-related infectious disease research.

### 2.2. Rotavirus Inoculum and Specific IgY

Human rotavirus (RVA/Human-tc/USA/Wa/1974/G1P [8]) was inoculated into cynomolgus monkeys at a dose of 3.1 × 10^7^ fluorescent focus-forming units (FFU) in 10 mL of solution as previously described [31]. The IgY used for this purpose was obtained from the eggs of immunised hens, as previously reported. An in vitro neutralisation assay was performed to evaluate antibody specificity and calculate the ideal concentration of IgY-neutralising activity to RVA (2.0 mg/mL) [29].

### 2.3. Study Design

Fourteen monkeys, identified via alphanumeric chest tattoos, were used in this study: the negative control (NC), J6, orally administered with 10 mL of isotonic saline solution; three positive controls (PC), B2, AE15 and AE11, orally administered with a 10 mL suspension of 3.1 × 10^7^ FFU human rotavirus Group A (RVA) Wa; two monkeys for the proof-of-concept (PoC), R9 and Q5, orally administered with 10 mL of RVA and 2.5 mg/mL of anti-RVA IgY previously incubated at 37 °C for 1 h and 30 min; four monkeys, AD15, AA3, AE5 and AE17, orally administered with 10 mL of RVA and, after 2 h, orally treated (OT) with 5 mL of anti-RVA IgY (2.5 mg/mL); and four monkeys, AD13, X5, AE1 and AE13, orally administered with 10 mL RVA and, two hours later, treated with 5 mL of anti-RVA IgY (2.5 mg/mL) orally and 1 mL of anti-RVA IgY (2.5 mg/mL) intravenously (OIVT). Polypropylene gavage tubes were used to deliver the inoculum directly into the stomach.

Blood and faecal samples were collected from inoculation (on day zero) onward. Clinical signs of diarrhoea (watery or semiliquid faeces), vomiting, ataxia, and dehydration were evaluated from the first to the fourth day post-inoculation (dpi). Body weight and temperature were recorded daily; fever was defined as a body temperature greater than 38.5 °C. Basal clinical pathology parameters were evaluated via a reference database of cynomolgus monkeys maintained under laboratory conditions [35]. Since the tropical environmental breeding colony could affect some basal parameters, haematological, biochemical, and virological data obtained at the nonhuman primate breeding colony in Fiocruz were used as baseline values. The animals were anaesthetised daily and subjected to clinical examinations and blood collection via the femoral vein [36]. At the fourth dpi, the animals were euthanised by total exsanguination under deep anaesthesia [36]. The experimental design is summarised in Table 1.

### 2.4. Haematological and Biochemical Analyses

A fully automated veterinary haematology analyser, pocH-100iVDiff (Sysmex Europe GmbH, Norderstedt, Schleswig-Holstein, Germany), was employed for complete blood count analysis. Potassium, chloride and sodium serum levels were analysed using a Vitros^®^250 chemistry analyser (Ortho-Clinical Diagnostics–Johnson & Johnson, Rochester, NY, USA).

### 2.5. Histopathological Analysis

Samples of the lung, liver, kidney, heart, stomach, mesenteric lymph nodes, spleen and random sections of the jejunum, ileum, and colon were fixed in 10% buffered formalin. The samples were dehydrated and embedded in Epredia™ histoplast paraffin, sectioned (4 µm) and stained with haematoxylin–eosin (H&E) and Giemsa [37]. Images were acquired with a Leica DM6 B digital microscope and Leica Application Suite X (LAS X—Leica Microsystems GmbH, Wetzlar, Hessen, Germany).

### 2.6. Immunofluorescence

Indirect immunofluorescence (IIF) assays were performed using fresh samples of intestinal tissue (jejunum and ileum) embedded in Tissue-Tek^®^ O.C.T. compound (Sakura Finetek, Torrance, CA, USA—4583). The tissues were frozen on dry ice, sectioned at 5 µm using a cryostat, mounted onto glass slides, and fixed in ice-cold acetone for 3 min. The tissue sections were washed with phosphate-buffered saline (PBS) at pH 7.2 and blocked at 37 °C for 3 h (4% bovine serum albumin (BSA), 0.1% Tween^®^ 20 and QS 50 mL of PBS at pH 7.2) in a humidity chamber. Then, the glass slides were washed three times in PBS (pH 7.2), incubated with primary antibodies overnight at 4 °C in a humidified chamber, and rewashed three times to remove unbound antibodies. The secondary antibody (conjugate) was added. After a 3 h incubation at 37 °C in a humid chamber, the glass slides were washed three times in PBS (pH 7.2) and counterstained with 1:20,000 Evans blue for 30 s. After three washes with PBS (pH 7.2), the slides were stained with 1:5000 4′,6-diamidino-2-phenylindole (DAPI) for 5 min. Images were captured with LSM META 510, LSM 710 confocal (Carl Zeiss, Jena, Thuringia, Germany) and AxioImager M1 fluorescence microscopes (Carl Zeiss) and analysed with Zen 2012 software (Carl Zeiss).

For IIF single staining of NSP4 and IFNγ, we used mouse IgG anti-NSP4 and rabbit IgG anti-IFNγ as primary antibodies, respectively. A donkey IgG anti-mouse/Alexa Fluor^®^ 488 conjugate was used as a secondary antibody for both NSP4 and IFNγ IIF staining. For IFF double staining with anti-CD4/anti-CD3 and anti-CD8/anti-CD3, we used mouse IgG anti-CD4, mouse IgG anti-CD8 and rat IgG anti-CD3 primary antibodies. The donkey IgG anti-mouse/Alexa Fluor^®^ 488 conjugate was used for CD4 and CD8 staining, and the goat IgG anti-rat/Alexa Fluor^®^ 647 conjugate was used for CD3 staining. The primary and secondary antibodies were diluted 1:100 and 1:1500, respectively.

### 2.7. RVA Detection and Quantification by RT–qPCR

Rotavirus A (RVA) detection and quantification by real-time polymerase chain reaction (RT–qPCR) were performed as previously reported [31]. RNA extraction from 10% faecal suspensions and serum samples was performed using the QIAamp Viral RNA Mini Kit^®^ (QIAGEN, Hilden, Germany —52906) following the manufacturer’s instructions. Reverse transcription was performed using the High-Capacity cDNA Reverse Transcription Kit (Applied Biosystems/Life Technologies, Carlsbad, CA, USA —4368813) via primers and probes to amplify the RVA VP6 gene [38]. RT–qPCR was performed using NSP3-specific primers and a TaqMan^®^ probe (Applied Biosystems/Life Technologies—4316034) as previously described [39] via the Applied Biosystems 7500 Real-Time PCR System (Applied Biosystems/Life Technologies —4366605). A standard curve was generated with serial dilutions (ranging from 10^0^ to 10^7^) of a previously characterised plasmid clone [40]. Samples that presented signals crossing the threshold line in both replicates until Ct ≤ 40 and presented a characteristic sigmoidal curve were considered positive. Target copy numbers were calculated on the basis of Ct values related to the standard curve. The number of copies per millilitre was determined by adjusting values according to the volume used for the nucleic acid extraction and qRT–PCR. The detection limit for this qRT–PCR assay is 4.4 × 10^2^ RNA copies/mg or mL [41].

## 3. Results

### 3.1. Clinical, Biochemical, and Histological Features

#### 3.1.1. Negative Control

**Figure 1:**
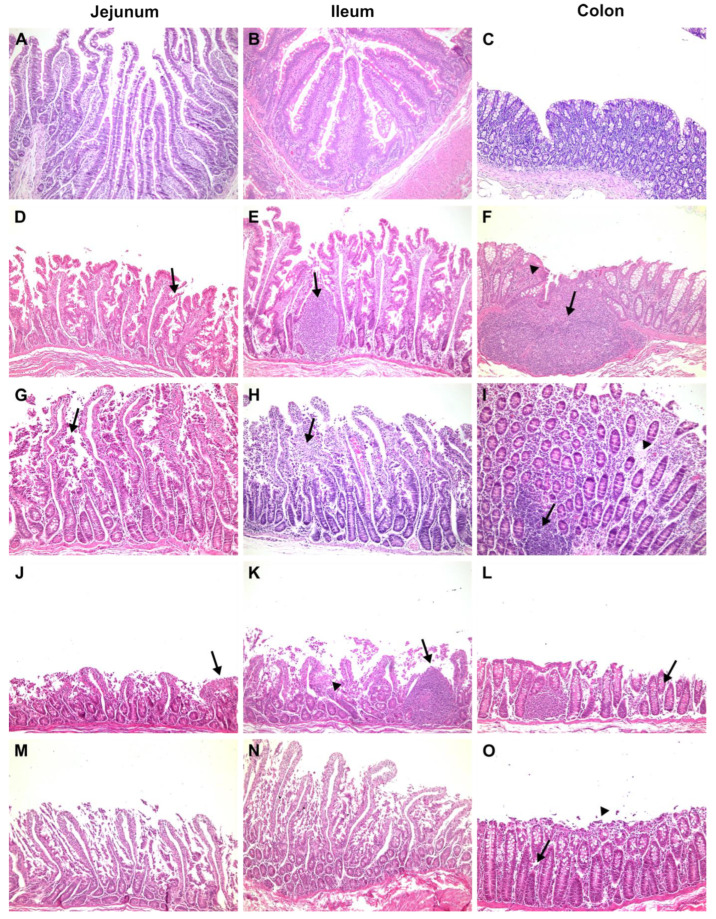
Histological analysis of the jejunum, ileum, and colon of the negative control (NC) (**A**–**C**), positive control (PC) (**D**–**F**), proof of concept (PoC) (**G**–**I**), orally treated (OT) (**J**–**L**), and orally and intravenously treated (OIVT) (**M**–**O**) cynomolgus monkeys. Images (×100 magnification) of the following H&E-stained tissue sections: jejunum: (**A**) normal villi height (NC); (**D**) decreased villi height, hyperplastic crypts and oedema in the lamina propria with desquamation (arrow) and loss of enterocytes (PC); (**G**) villous shortening, epithelial vacuolation, desquamation, and loss of enterocytes (arrow) (PoC); (**J**) reduction in the villus-to-crypt length ratio, villus loss and fusion at the tips of villi (arrow) (OT); (**M**) reduction in the villus-to-crypt length ratio, villus loss and fusion (OIVT). Ileum: (**B**) normal villus height (NC); (**E**) irregular villous architecture and height, with crypt hyperplasia (arrow) (PC); (**H**) highly vacuolated villus, with oedema and coagulation necrosis in the middle and upper regions of villi (arrow) (PoC); (**K**) severe villus atrophy and fusion, with cellular debris in the lamina propria (arrow head), and nodular lymphoid hyperplasia (arrow) limited to the lamina propria (OT); (**N**) loss of villous enterocytes with necrosis and sloughing of the degenerate epithelial barrier (OIVT). Colon: (**C**) normal mucosa with a relatively high goblet-to-absorptive cell ratio (NC); (**F**) denudation of the epithelial mucosa, massive lymphoid aggregation (arrow), and dense infiltration of the lamina propria with mononuclear cells (arrow head) (PC); (**I**) sloughing of epithelial cell surfaces and moderate oedema of the lamina propria (arrow head), with lymphoid nodules (arrow) (PoC); (**L**) thinning of the colon mucosa with epithelial desquamation (arrow) (OT); (**O**) desquamated and necrotic epithelium (arrow head) and focal sloughing of sheets of epithelium, with oedema of the lamina propria (arrow) (OIVT).

**Figure 2:**
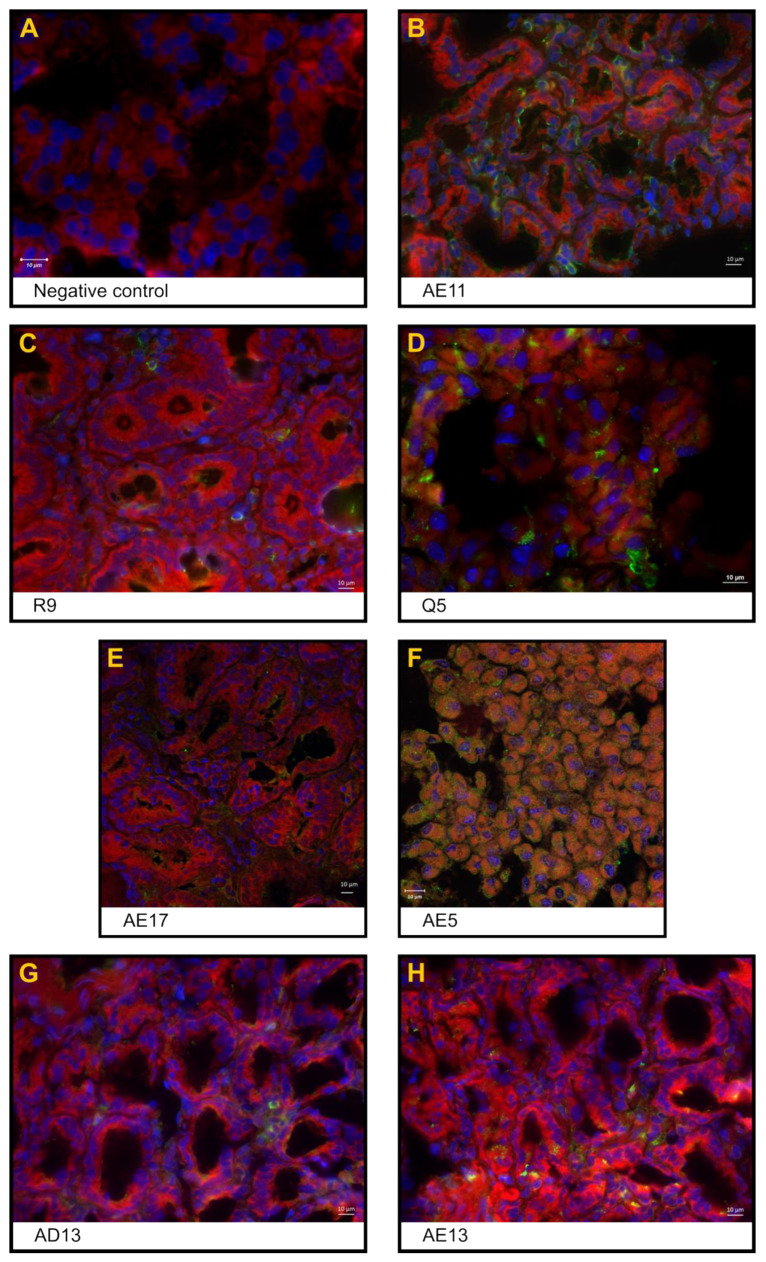
Indirect immunofluorescence images of jejunum sections from RVA-inoculated cynomolgus monkeys. (**A**) Negative control: absence of fluorescence; (**B**) positive control, AE11: NSP4+ epithelial cells (enterocytes); (**C**) proof of concept, animal R9: NSP4+ cells in the inflammatory infiltrate of the interstitial space of Lieberkühn crypts; (**D**) proof of concept, animal Q5: NSP4+ cells in enterocytes; (**E**) orally treated AE17 monkey: NSP4+ cells in epithelial cells from the Lieberkühn crypt; (**F**) orally treated AE5 monkey: NSP4+ cells in lamina propria from the crypt area; (**G**) orally and intravenously treated AD13 monkey: NSP4+ cells in the interstitial space of the crypt zone; (**H**) orally and intravenously treated AE13 monkey: NSP4+ infiltrating cells in the interstitial space of the crypt zone. NSP4 staining inside enterocytes from the crypt region. Anti-NSP4 antibody (Alexa Fluor^®^ 488–green) was used; nuclei were stained with DAPI (blue), and parenchyma was stained with Evans blue (red).

The negative control (code-named J6), orally administered with 10 mL of isotonic saline solution, had no diarrhoea, no food intake variation, and no viral shedding detected in faeces throughout the study. Haematological and biochemical parameters are normally related to age. The jejunum, ileum and colon were histologically unaffected. Figure 1A,B shows enterocytes covering the luminal surface of the intestinal villi. The mucosa of the colon is lined by a simple columnar epithelium with a thin brush border and numerous goblet cells. The crypts of Lieberkühn are straight and unbranched and lined with goblet cells. In the lamina propria, leukocytes are abundant (Figure 1C). NSP4 protein expression (Figure 2), CD29+, CD3+CD4+, and CD3+CD8+, and IFN-gamma+ cells were not detected.

#### 3.1.2. Positive Control

The cynomolgus monkeys AE11, AE15 and B2, which were orally administered 10 mL of human rotavirus suspension (3.1 × 10^7^ FFU RVA Wa), had no signs of diarrhoea, emesis, weight loss, or fever throughout the study. Monkey AE15 showed eosinophilia at 1 dpi. Cynomolgus B2 presented with hypochromic normocytic anaemia at 4 dpi, leucocytosis at 1 dpi with discreet eosinophilia and hyperkalaemia at 4 dpi. No haematological or biochemical alterations were observed. Rotavirus enteritis leads to morphometric changes in the jejunal and ileal mucosa. Morphological analysis revealed a reduction in the villus-to-crypt length ratio, villus denudation, epithelial vacuolation and desquamation, and loss of enterocytes from the villi (Figure 1D,E). The lamina propria from villi and crypts was expanded (oedema), with infiltrated plasmocytes, eosinophils, neutrophils, and macrophages. The colon revealed crypt architectural distortion associated with interstitial oedema and moderate mononuclear cell infiltration within the lamina propria. A mucosa with a lymphoid follicle containing a well-formed germinal centre that extends to the submucosa was observed (Figure 1F). Rotavirus nonstructural protein 4 (NSP4) expression was localised around the crypts (Figure 2), where inflammatory mononuclear cells were grouped, as in the absorptive surface of epithelial cells (AE11). CD29+, CD3+CD4+, CD3+CD8+, and IFN-gamma+ cells were detected in the lamina propria of villi and crypts. Animal AE11 had RNA detected in faeces.

#### 3.1.3. Proof of Concept

One infected animal, Q5, showed clinical signs of RVA infection, such as severe diarrhoea and anorexia, during the four days of the study; however, it never met the requirements of the humanitarian endpoint during the clinical examination according to the NHP guidelines [33]. Both the Q5 and R9 haematological analyses revealed leucocytosis and neutrophilia from 2 to 3 dpi; Q5 had hypochloraemia from 1 to 4 dpi and decreased sodium levels. The jejunum and ileum showed histopathological changes, such as villi detachment and apoptotic debris, and shed enterocytes into the lumen (Figure 1G,H). The colonic mucosa showed denuded epithelium surfaces and oedema due to inflammatory infiltration of lymphocytes and plasma cells in the lamina propria (Figure 1I). Immunofluorescences for NSP4+ (Figure 2), IFN gamma+, and CD29+ cells and eosinophils were detected around the crypts, whereas T lymphocytes were detected in the lamina propria of the villi. RVA RNA was detected in the stool samples of both animals. Despite biochemical disorders, R9 did not show clinical signs of gastroenteritis.

#### 3.1.4. Cynomolgus Monkeys Orally Treated with Anti-RVA IgY

Monkeys AE5 and AD15 presented monocytosis at 1 and 3 dpi, respectively, with severe lymphopenia associated with hyperkalaemia at 4 dpi. AE17 presented eosinophilia at 1 to 2 dpi with monocytosis at 1 dpi and leucocytosis at 2 dpi. However, none of the orally treated monkeys, AD15, AA3, AE5 or AE17, presented clinical signs of rotavirus infection (fever, diarrhoea, and anorexia).

Histological lesions were identified in the small bowel as villous shortening, epithelial vacuolation and desquamation, and loss of enterocytes from the villi. Increased goblet cell density was associated with epithelial tumefaction (Figure 1J,K). Monkey AE17 exhibited abnormal thinning of the colonic mucosa with epithelial desquamation (Figure 1L). Infected cells were assessed by immunofluorescence for NSP4+ in the villi and small intestinal crypts (Figure 2). CD3+CD4+ and CD3+CD8+ cell infiltration was scattered throughout the lamina propria of the villi. CD29+ cells were detected in interstitial cells and on the apical side of epithelial cells. Viral RNA was detected in rectal samples at 1 to 2 dpi.

#### 3.1.5. Cynomolgus Monkeys Orally and Intravenously Treated with Anti-RVA IgY

Monkey X5 had eosinophilia and lymphocytosis detected at 1 dpi onward; AE1 had lymphocytosis and eosinophilia observed at 1 and 4 dpi, respectively. Monkey X5 developed diarrhoea, and AE13 presented fever and anorexia. No other signals of rotaviruses and serum electrolytes were detected.

The histological lesions in the intestines included a decreased villus-to-crypt length ratio, loss of villi, and widespread loss of the intestinal epithelium (Figure 1M,N). Syncytial cells, histocytes, and lymphocytes were detected in the lamina propria because of interstitial enteritis. Epithelial denudation of colonic mucosa mononuclear cell debris and desquamated epithelial cells were detected in the colon lumen (Figure 1O). NSP4+ infiltrating cells were observed in the interstitial space of the crypt zone (Figure 2), and CD3+CD4+, CD3+CD8+ and CD29+ cells were detected in the lamina propria of the villi.

Table 2 summarises the changes in the small intestine, revealing the severity of villous denudation and cellular interstitial infiltration in the jejunum and ileum. Diarrhoea, enteritis, and colitis are associated with viral RNA shedding. These histological changes were not observed in the other groups of cynomolgus monkeys in this study.

### 3.2. Phenotypic Analysis of the Inflammatory Infiltrate in the Small Intestine

**Figure 3:**
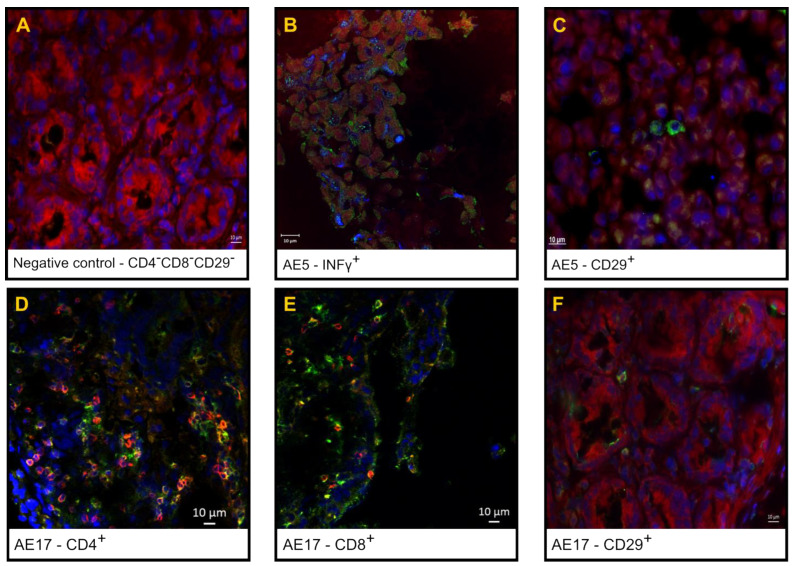
Indirect immunofluorescence (IIF) images of IFN-γ, CD29^+^, CD3^+^CD4^+^ and CD3^+^CD8^+^ T lymphocytes in jejunum sections from the negative control, positive control, and orally treated RVA-inoculated cynomolgus monkeys. Negative control: shaded area (**A**); orally treated, animal AE5: IFN-gamma-positive cells (**B**); CD29^+^ T cells in jejunum tissue section (**C**); orally treated, animal AE17: anti-CD4/Alexa Fluor^®^ 647 antibody (red), anti-CD4/FITC (green), and DAPI-stained nuclei (blue), (×400) (**D**); anti-CD8/Alexa Fluor^®^ 647 antibody (red), anti-CD4/FITC (green) and DAPI-stained nuclei (blue) (**E**); and CD29^+^ T cells in jejunum tissue section (**F**).

Figure 3 shows the double staining and single staining of CD3+/CD4+ T lymphocytes in inflammatory infiltrates, as shown by confocal laser scanning immunofluorescence microscopy of the interstitial area of the villi and Lieberkühn crypts of the jejunum tissue sections from a cynomolgus monkey orally treated with anti-RVA IgY and the negative and positive controls.

### 3.3. Expression of the Rotavirus Nonstructural Protein NSP4 in Enterocytes and Inflammatory Cells in the Interstitial Space

The expression of the RVA replication marker NSP4 was found in the jejunum of all inoculated animals, even those treated with anti-RVA IgY, predominantly in the cell membrane of polarised epithelial cells (enterocytes) localised in Lieberkühn crypts and inflammatory cells detected in the interstitial space (lamina propria) (Figure 2).

### 3.4. Viral RNA Shedding

Except for animal AD13, all animals had RVA RNA detected in faeces within one to two or one to three dpi (AE15) without intermittence. Viral RNA was not detected in the serum samples of any of the animals.

## 4. Discussion

Both wild and captive cynomolgus monkeys are naturally susceptible [42] and exhibit the clinical features of the human RVA infection [43]. Cynomolgus individuals orally inoculated with the RVA Wa prototype presented nonspecific signs of human rotavirus infection, such as vomiting and diarrhoea, occasionally accompanied by low-grade fever and dehydration [44]. Therefore, as shown in a previous study by our group [31], the cynomolgus is a worthy model for studying rotavirus-induced inflammation of the small bowel. In the present study, all animals inoculated with human RVA Wa, not treated or treated with anti-RVA IgY, exhibited damage-associated intestinal inflammation. Although elderly animals are considered less susceptible to rotavirus infection [45], the positive control in our study, a 27-year-old monkey, developed severe histological enteritis.

The degree of viral shedding in the cynomolgus model is generally lower than that in human RVA-induced acute gastroenteritis (10^10–11^ genomic copies/mg) [46]. In our study, all but one RVA-inoculated monkey exhibited viral shedding; most exhibited very low viral loads. A 5-year-old monkey (young adult), orally administered anti-RVA IgY, presented the highest faecal viral load (1.40 × 10^4^ copies/mg) at the first dpi. This low degree of viral shedding occurs because macaques are less susceptible to human RVA heterologous infections, as shown in rhesus monkeys that are naturally infected [47]. In a previous study, RVA was detected in the faeces of inoculated monkeys on the first to third dpi and was related to the inoculum. After one or two days without viral excretion, a second instance of viral shedding was detected at the 5th to 10th dpi, coincident with viral replication in the intestine [31]. The second RVA shedding could not be observed in the present study. However, viral replication of the heterologous RVA Wa was confirmed by NSP4 release from cryptal cells of the small intestine of inoculated monkeys. The rotavirus NSP4 protein is a marker of replication and virulence [48] and is cytotoxic when it is transiently expressed in cells by inducing secretory diarrhoea [49]. Although the present study did not assess the duration of viral shedding in cynomolgus monkeys, we assume that diarrhoea should not be considered the gold standard for RVA infection in this animal model. However, this study aimed to histologically confirm the protective effect of IgY in the small intestine of treated monkeys.

RVA tropism to the small bowel was confirmed in this study, as demonstrated by the presence of mucosal inflammatory injury in all inoculated cynomolgus monkeys. Previous studies reported similar results in rhesus monkeys [50] and mice [51]. Jain and Haydel (2023) recently described mucosal inflammatory injuries in children infected with RVA [52]. Histopathological colon changes in the absence of diarrhoea were observed in 5 of the 13 inoculated animals in this study, as also described in human RVA infections [53,54].

Although this study could not assess the humoral response, the inoculated monkeys exhibited an intestinal immune-inflammatory response after RVA challenge. As evidenced by May–Grunwald Giemsa staining and indirect immunofluorescence, eosinophils, plasmocytes, and other lymphocytes (CD3+CD4+ and CD3+CD8+), including immune activation markers such as CD29+ and IFN-γ+ cells, were localised in the upper third, middle third, or whole villi, thus suggesting that inflammatory cells migrated from the lamina propria and crypts of Lieberkühn’s interstitial space. Large foamy macrophages and abundant intracytoplasmic phagocytic material were also localised in the lamina propria. Reactive lymphoid follicles (Peyer’s patches) with multinucleated syncytial cells were frequently detected in the gut-associated lymphoid tissues at the ileal mucosa. Goblet cells (mucin-producing cells) and proliferative Paneth cells, which play significant roles in preventing viral inflammatory injury and repairing the intestinal epithelial layer [55], were also detected in the crypts of Lieberkühn.

In animals with severe virus-induced enteritis (B2, Q5, AE17, X5, and AE1), crypt injury may fragment the myofibres of the muscularis mucosae. The abundant and heterogeneous debris (exudative enteritis) suggests that epithelial cell desquamation is associated with RVA-induced apical villus injury. One monkey that was orally inoculated with IgY previously neutralised with RVA (proof-of-concept), which died overnight due to severe dehydration and electrolytic loss, was diagnosed with exudative enteritis at necropsy. Notably, differential overexpression of NSP4+ enterocytes was detected in the small intestine of this animal. Recent studies have demonstrated that the NSP4 VPD is a Ca^2+^-conducting viroporin, a mechanism by which NSP4 disturbs enterocyte Ca^2+^ homeostasis [56]. The intestinal villi secrete chloride as a result of rotavirus infection. The chloride secretory response is regulated by a phospholipase C-dependent calcium signalling pathway induced by NSP4 [57]. Monkey Q5 was the only animal that exhibited a decrease in both chlorine and sodium levels. The other animals did not show considerable variations in electrolyte dosages. The proof-of-concept group aimed to see if the antibody was able to neutralise the virus, and it did not infect the animals. The antibody may have dissociated when it passed through the animal’s stomachs and the virus was free to internalise into the cells.

Human rotavirus may infect differentiated enterocytes and enteroendocrine cells, with consequent viroplasms and lipid droplet induction, as demonstrated by in vitro studies with human intestinal enteroid cell culture. Human rotavirus infection, as a rotavirus enterotoxin treatment for human intestinal enteroids, promoted physiological luminal expansion, thus confirming virus-induced fluid secretion and diarrhoea [58].

In our study, which was designed to evaluate histopathological changes in the small intestine in cynomolgus monkeys of different ages, diarrhoea was not a clinically evident marker of rotavirus-induced tissue damage, reinforcing our previous results [31]. Diarrhoea was observed in only two monkeys, which does not represent an established standard for challenging antivirus drugs, vaccines, and antibody therapy against acute rotaviruses. Similarly, in rotavirus-infected patients, diarrhoea can occur without visible tissue damage; conversely, histological lesions can be asymptomatic [57]. In another study, five of six cynomolgus monkeys (newborns) orally inoculated with human rotavirus developed diarrhoea. Faecal shedding of RVA inoculated in the other six newborn monkeys induced diarrhoea in only one animal [59]. Antibody neutralisation of intestinal/luminal-free RVA has been considered a therapeutic approach for immunocompetent children with acute RVA-induced gastroenteritis. It significantly reduced the duration of diarrhoea by 76 h [10]. In immunocompromised children with chronic rotavirus diarrhoea, oral immunoglobulin administration resulted in effective RVA-neutralising action in all three patients [60].

The histological findings of this study suggest that our anti-RVA IgY conferred protection against rotavirus-induced enteritis in four of the ten treated monkeys, which presented a normal intestinal mucosa. The low efficacy of our anti-RVA IgY is probably due to in vivo neutralisation in the gastric environment [61]. There are, indeed, some studies demonstrating that the use of microcapsules could protect IgY against digestive inactivation [62,63,64]. Additionally, to some extent, the chicken ovomucoid protein protects IgY antibodies against acid proteolytic degradation [64,65]. The oral administration of IgY prevented bovine rotavirus-induced diarrhoea in newborn calves [16]. Similar protection was conferred against human rotavirus-induced diarrhoea in neonatal gnotobiotic pigs prophylactically treated with human RVA-specific IgY antibodies [17]. An improved therapeutic effect against rotavirus-induced diarrhoea was observed in infant mice treated with increased doses of immunoglobulin [66]. Our data reinforce that polyclonal IgY could be helpful as an incremental therapy for treating rotavirus enteritis, as already reported [16,17,28,66]. The principal mode of action is the binding of antibodies to viral capsid proteins. The hypothesis is that these proteins can be easily recognised by antibodies and that this binding can lead to a failure of the biological functions of these components, which play important roles in binding to intestinal cells, cell internalisation and viral replication. In this way, antibodies protect and prevent the invasion of epithelial cells. At the same time, one possibility would be less elimination of infectious particles in the faeces, since the antibody would be bound to the viruses, thereby neutralising them.

Although diarrhoea is considered the gold standard for evaluating the efficacy of therapeutic approaches for treating rotavirus gastroenteritis, it could not be addressed in our study since only two animals presented this clinical sign. The present study showed that histopathological assessment should serve as a reference for monitoring rotavirus infection outcomes in this animal model. The main goal of this study was to describe the histological features of RVA-induced enteritis in a cynomolgus model, regardless of the presence of clinical signs. Although immunotherapy with IgY did not prevent viral shedding in nine of the ten treated monkeys, anti-RVA IgY had a protective effect against severe enteritis in four of the ten treated animals, as evidenced by histopathological findings.

## Figures and Tables

**Table 1:** Experimental design for cynomolgus rotavirus Group A (RVA) infection.

	Cynomolgus Code Name	Age (Years/Months)	Weight (kg)	Inoculum Dose (FFU ^a^)	IgY (mg)
Negative control (NC)	J6	19/01	3.380	Ø	Ø
Positive control (PC)	B2	27/00	3.650	3.1 × 10^7^	Ø
	AE15	0/10	1.330		
	AE11	1/00	1.340		
Proof of concept (PoC)	R9	11/08	5.840	3.1 × 10^7^	2.5 (oral)
	Q5	13/02	4.500		
Oral immunotherapy (OT)	AD15	1/10	1.600	3.1 × 10^7^	2.5 (oral)
	AA3	5/03	3.970		
	AE5	1/02	1.250		
	AE17	0/10	1.130		
Oral and intravenous immunotherapy (OIVT)	AD13	1/11	1.150	3.1 × 10^7^	2.5 (oral) 2.5 (IV ^b^)
	X5	7/03	6.650		
	AE1	1/03	1.550		
	AE13	1/00	1.200		

^a^ FFU: fluorescent focus-forming unit. ^b^ IV: intravenous. Ø: Not administered.

**Table 2:** Immunoglobulin IgY treatment outcomes in cynomolgus monkeys experimentally infected with rotavirus: Group A: histological features of the intestinal mucosa, viral shedding, rotavirus nonstructural protein detection, and rotavirus-associated diarrhoea.

	Monkey	Villi Denudation ^a^		Histological Enteritis	Interstitial Cell Infiltration ^b^		Colitis	RVA Shedding (RNA Copies/mg) ^c^	NSP4 ^d^	Rotavirus-Associated Diarrhoea	Outcome ^e^
		Jejunum	Ileum		Jejunum	Ileum					
NC	**J6**	Ø	Ø	Ø	Ø	Ø	no	no	ND	no	control
PC	**B2**	†	†††	severe	†	†	ND ^f^	no	ND	no	sick
	**AE15**	††	Ø	moderate	†††	††	no	10^2^ (1 dpi)	ND	no	sick
	**AE11**	††	†	moderate	†	†	yes	10^3^ (1 dpi)	††	no	sick
PoC	**R9**	Ø	Ø	mild	Ø	Ø	no	10^3^ (2 dpi)	†	no	protected
	**Q5**	††††	††††	severe	†††	†††	yes	10^3^ (2 dpi)	†††	yes	sick/death ^g^
OT	**AD15**	Ø	†	mild	†	†	ND	10^3^ (2 dpi)	ND	no	protected
	**AA3**	†	†	mild	†	†	no	10^4^ (1 dpi)	ND	no	protected
	**AE5**	†	†††	moderate	Ø	Ø	no	10^3^ (2 dpi)	††	no	sick
	**AE17**	††††	††††	severe	††	††	yes	10^3^ (2 dpi)	†	no	sick
OIVT	**AD13**	†	†	mild	Ø	Ø	no	no	††	no	protected
	**X5**	†††	†††	severe	††	††	no	10^3^ (2 dpi)	ND	yes	sick
	**AE1**	†††	†††	severe	††	††	yes	10^2^ (1 dpi)	ND	no	sick
	**AE13**	††	††	moderate	†††	††	yes	10^3^ (2 dpi)	†††	no	sick

^a^ Villous denudation: Ø—absent; †—apical; ††—medium; †††—basal; ††††—crypt. ^b^ Interstitial infiltration of cells: Ø—absent; †—mild; ††—moderate; †††—severe. ^c^ Maximum viral load detected and days post-inoculation (dpi). ^d^ NSP4: †—mild; ††—moderate; †††—severe. ^e^ The animal was classified as “sick” when positively diagnosed by histological small bowel lesions. ^f^ ND—not done. ^g^ Animal Q5 died in the early morning during the clinical examination (4 dpi).

## Data Availability

The original contributions presented in the study are included in the article, further inquiries can be directed to the corresponding author.
